# Risk factors for postpartum sepsis: a nested case-control study

**DOI:** 10.1186/s12884-020-02991-z

**Published:** 2020-05-14

**Authors:** Samina Bakhtawar, Sana Sheikh, Rahat Qureshi, Zahra Hoodbhoy, Beth Payne, Iqbal Azam, Peter von Dadelszen, Laura Magee

**Affiliations:** 1 Department of Community Health Sciences, Aga Khan University, Karachi, Pakistan; 2 Department of obstetrics and gynecology, Aga Khan University, Karachi, Pakistan; 3 University of British Columbia, Vancouver, Canada; 4 Global Women’s Health, King’s College, London, UK

**Keywords:** Sepsis, Risk factors, Postpartum women, Lower-middle income country

## Abstract

**Background:**

The Majority (99%) of maternal deaths occur in low and middle-income countries. The three most important causes of maternal deaths in these regions are postpartum hemorrhage, pre-eclampsia and puerperal sepsis. There are several diagnostic criteria used to identify sepsis and one of the commonly used criteria is systematic inflammatory response syndrome (SIRS). However, these criteria require laboratory investigations that may not be feasible in resource-constrained settings. Therefore, this study aimed to develop a model based on risk factors and clinical signs and symptoms that can identify sepsis early among postpartum women.

**Methods:**

A case-control study was nested in an ongoing cohort of 4000 postpartum women who delivered or were admitted to the study hospital. According to standard criteria of SIRS, 100 women with sepsis (cases) and 498 women without sepsis (controls) were recruited from January to July 2017. Information related to the socio-demographic status, antenatal care and use of tobacco were obtained via interview while pregnancy and delivery related information, comorbid and clinical sign and symptoms were retrieved from the ongoing cohort. Multivariable logistic regression was performed and discriminative performance of the model was assessed using area under the curve (AUC) of the receiver operating characteristic (ROC).

**Results:**

Multivariable analysis revealed that 1–4 antenatal visits (95% CI 0.01–0.62).

, 3 or more vaginal examinations (95% CI 1.21–3.65), home delivery (95% CI 1.72–50.02), preterm delivery, diabetes in pregnancy (95% CI 1.93–20.23), lower abdominal pain (95% CI 1.15–3.42)) vaginal discharge (95% CI 2.97–20.21), SpO2 < 93% (95% CI 4.80–37.10) and blood glucose were significantly associated with sepsis. AUC was 0.84 (95% C.I 0.80–0.89) which indicated that risk factors and clinical sign and symptoms-based model has adequate ability to discriminate women with and without sepsis.

**Conclusion:**

This study developed a non-invasive tool that can identify postpartum women with sepsis as accurately as SIRS criteria with good discriminative ability. Once validated, this tool has the potential to be scaled up for community use by frontline health care workers.

## Background

Pregnancy and childbirth-related complications are a major public health concern worldwide. Approximately 810 women globally die every day from preventable causes related to pregnancy and childbirth and almost one-third of these occur in South Asia [1]. About 60% of maternal deaths occur during delivery and the immediate postpartum period [2]. In Pakistan, there is one death every 40 min due to pregnancy or delivery complications [3]. Pakistan Demographic Health Survey (PDHS 2006) reported sepsis as a third major cause of maternal mortality which contributes to 14% of maternal deaths in Pakistan [4, 5]. According to International Consensus, sepsis is defined as “life-threatening organ dysfunction caused by a dysregulated host response to infection” [6]. Increasing severity of infection correlates with increased mortality, which is 16.7% for sepsis, 25–30% for severe sepsis and up to 40–70% for septic shock [7, 8] in the general population.

There are many associated distant, intermediate and proximal risk factors contributing to sepsis. Distant and intermediate factors are those which make women vulnerable or predispose them to develop sepsis. Our primary concern is proximal risk factors which can lead to sepsis within few hours and provide a window of opportunity to identify women at high risk of sepsis [9].

Previous studies on sepsis were focused on sepsis in the general population but limited studies have taken into account physiological changes of pregnancy and the postpartum period. Catherine et al., in 2011, designed sepsis obstetric score (SOS) among pregnant and postpartum women in emergency department to identify the risk of intensive care unit (ICU) admission. This scoring system took into account clinical parameters and laboratory investigations and reported sensitivity and specificity of 88.9 and 95.2% respectively (AUC ROC = 0.92) [10]. The limitation of this obstetric score was that it involved immature neutrophils and serum lactate levels which are not feasible in low resource settings.

The progression of sepsis is lethal hence early identification may help reduce further complications. The study aimed to develop a model based on risk factors and clinical sign and symptoms that can enhance early identification of postpartum women with sepsis in low resource settings.

## Methods

A large cohort study on 4000 postpartum women aged 15–49 years has been taken place at Jinnah Postgraduate Medical Center (JPMC) from October 2016 to May 2017 to develop a predictive model to identify women with severe maternal outcomes following childbirth.

This case-control study was nested on the larger cohort. The study was conducted at (JPMC) from January to May 2017 to determine risk factors and clinical sign and symptoms for identification of sepsis among postpartum women. JPMC is one of the largest public health facilities in Karachi which serves a large catchment area within and outside Karachi, representing a diverse patient population. Women were screened through a structured questionnaire to ensure the fulfillment of inclusion and exclusion criteria. The case is defined as postpartum women who have term delivery and have sepsis whereas, controls were women with term delivery without sepsis. Sepsis was defined as women who fulfilled two of four criteria according to SIRS criteria. These included heart rate > 90beats/ minute, respiratory rate > 20 breaths/minutes, temperature > 38.0 C or < 36.0 C and white blood cell counts > 12,000/mm^3^ and < 4000/mm^3^ [11]. Postpartum women with any autoimmune diseases, having any previous admission to any hospital, unable to provide informed consent in Urdu and those who had missing information or incomplete follow up from the cohort were excluded from the study. The exclusion criteria were similar for both cases and controls. Total 598 postpartum women were eligible to participate in this study and all were above 18 years of age.

Data was collected by medical doctors and nurses who were hired for the research study and received one-day training for the data collection. The research team collected information on demographics (e.g. education, occupation, household income and assets), antenatal care and use of tobacco through interview using a structured pre-tested questionnaire. Blood pressure, blood oxygen saturation level (SpO2) and blood glucose level were objectively measured with validated the point of care devices. The information regarding comorbid such as hypertension and diabetes, index pregnancy and delivery details and vital (temperature, respiratory rate, heart rate) were collected through medical records. For assuring data integrity weekly meeting conducted to observe the status, quality and issues in collecting data that has been endured by the research team.

**Fig. 1:**
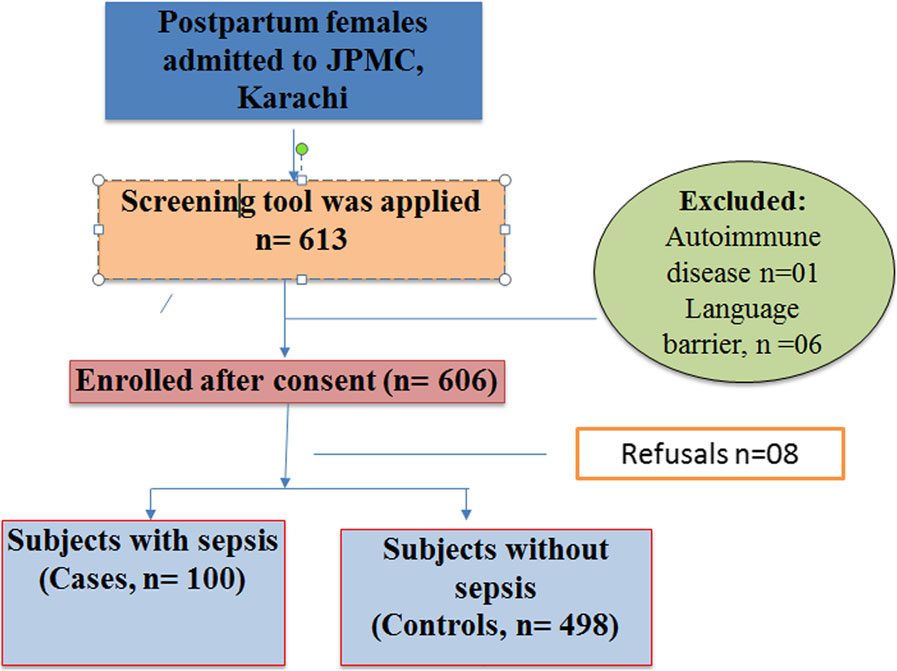
Participant flow chart

Before administering study questionnaire, the study was explained to the patients and written informed consent was obtained. If participant was not educated, then thumb impression was taken along with a witness signature. All participants were above 18 years of age so no parental consent was required (Fig. 1). The sample size calculation for this nested case-control study was performed using Open EPI version 3.1. minimum sample size of 100 cases and 498 controls was required in order to achieve 80% power, with an anticipated prevalence of risk factors among the controls ranging from 4 to 59%, an anticipated odds ratio of 2 and a level of significance of 5%.

Means and standard deviation were estimated for normally distributed continuous data and proportions for the categorical variables. WAMI (Water, assets, maternal education and household income) scoring system was used for formulating socioeconomic status based on monthly income, education and household assets [11] (Table 1). Odds ratio (OR) with 95% confidence interval (CI) were computed using binary logistic regression analysis. Based on our literature review, socioeconomic status and mode of delivery were considered as potential confounders but the variables were statistically insignificant and did not qualify as confounders. Hence, the final model was not adjusted for these variables. Model calibration was assessed by Hosmer Lemeshow test and model accuracy was assessed by receiver operating curve (ROC) by plotting sensitivity against 1- specificity for different cut-offs of parameters. All statistical analysis was performed using STATA version 12.

**Table 1:** Descriptive of variables

Baseline characteristics of the study participants			
Variables	Cases *n* = 100 n (16.7%) Mean ± SD	Controls *n* = 498 n (83.27%) Mean ± SD	*P* value
**Socio-demographic Information:**			
Maternal age	27.1 ± 5.10	26.6 + 5.02	0.41
Socioeconomic status			
Low tertile	35 (35.00)	163 (32.30)	0.88
Middle tertile	39 (39.00)	198 (40.00)	
High tertile	26 (26.00)	137 (27.70)	
**Pregnancy and delivery Information:**			
Booking status			
No	21 (21)	73 (14.66)	0.12
Yes	79 (79)	425 (85.36)	
Antenatal care visits			
0 times	14 (14)	47 (9.44)	
1–4 times	21 (21)	223 (44.76)	
> 4 times	65 (65)	228 (45.70)	0.04
Antenatal care provider			
None	14 (14)	47 (9.44)	
Skilled Birth attendant	84 (84)	445 (89.36)	0.47
Unskilled birth attendant	2 (2)	6 (1.26)	
Parity			
0	1 (1)	3 (0.60)	0.65
1–4	84 (84)	431 (86.55)	
> 5	15 (15)	64 (12.85)	
Mode of delivery			
Spontaneous vaginal	58 (58)	293 (58.84)	
delivery	3 (3)	24 (4.82)	0.73
Assisted delivery	39 (39)	181 (36.3)	
Caesarean delivery			
Prolonged labor			
< 12 h	84 (84)	428 (85.92)	0.58
> 12 h	16 (16)	70 (14.10)	
Rupture of membrane			
< 24 h	97 (97)	478 (95.98)	0.50
> 24 h	3 (3)	20 (4.0)	
Number of vaginal examination			
1–3 times	54 (54)	348 (69.86)	0.002
> 3 times	46 (46)	150 (30.10)	
Place of delivery			
Health facility	94 (94)	494 (99.2)	
Home	6 (6)	4 (0.80)	0.001
Preterm			
No	80 (80)	443 (88.9)	0.019
Yes	20 (20)	55 (11.01)	
Diabetes in pregnancy			
No	90 (90)	488 (97.9)	0.005
Yes	10 (10)	10 (2.01)	
**Clinical Sign and Symptoms:**			
Upper abdominal pain			
No	94 (94)	472 (94.7)	0.26
Yes	6 (6)	26 (5.21)	
Lower abdominal pain			
No	44 (44)	303 (60.84)	< 0.001
Yes	56 (56)	195 (39.12)	
Vaginal discharge			
No	86 (86)	481 (96.55)	< 0.001
Yes	14 (14)	17 (3.41)	
Dyspnea			
No	93 (93)	471 (94.50)	0.54
Yes	7 (7)	27 (5.42)	
Spo2			
> 93%	81 (81)	488 (98.10)	< 0.001
< 93%	19 (19)	9 (1.81)	
Blood glucose	96.5 + 15.6	110.8 + 34.00	0.004
Systolic blood pressure	110.1 + 12.2	116.1 + 13.45	0.5
Diastolic blood pressure	74.9 + 9.9	74.6 + 10.30	0.32

## Results

The mean age of the cases was 27.1 +/− 5.10 and of controls was 26.6 +/− 5.02. The majority of women in cases (40%) and controls (39%) belonged to the middle tertile of socioeconomic status. Cases had higher proportion of more than 3 vaginal examinations (46%) and cesarean deliveries (39%) as compared to controls (36 and 30% respectively). Preterm delivery was present in 20% of women with sepsis as compared to 11% in controls.

Proportions of women reporting lower abdominal pain, vaginal discharge and dyspnea were more common among cases as compared to controls (56, 14 and 7% respectively). Approximately one-fifth (19%) of cases were found to have a significantly low oxygen saturation of < 93% as compared to controls (1.81%) (*p* value = 0.001). Mean blood glucose among cases was 96.5 +/− 15.6 mg/dl while it was 110.9 +/− 34.0 mg/dl in controls. (Table 1).

In this study women receiving 1–4 antenatal visits were 75% less likely in women with sepsis versus women without sepsis (aOR 0.25, 95% CI0.01–0.62). Increased number of vaginal examinations was 2 times higher among cases as compared to controls (aOR 2.10; 95% CI = 1.21–3.65). Home delivery was approximately 9 times more likely in cases as compared to controls (95% CI = 1.72–50.02).

Preterm delivery was 3.15 times (95% CI = 1.58–6.25) higher among women with sepsis as compared to those without sepsis. Cases were also more likely to be diabetic (aOR 6.62 (95% CI = 1.93–20.23)) than controls. The odds of lower abdominal pain and vaginal discharge was high among cases as compared to controls (aOR1.99 (95% CI = 1.15–3.42); (aOR7.77 (95% CI = 2.97–20.21). The odds of low oxygen saturation < 93% was 13 times high in septic cases as compare to controls (aOR = 13.0; 95% CI = 4.80–37.10). The final model was presented including information related to pregnancy and delivery **(**Table 2)**.**

**Table 2:** Model based on risk factors and clinical signs and symptoms

Variables	Unadjusted OR (95% confidence interval)	Adjusted OR (95% confidence interval)
**Pregnancy and Delivery Information:**		
Antenatal visits		
None (reference)	–	–
1–4	0.31 (0.14–0.66)	0.25 (0.01–0.62)
> 4 visits	0.95 (0.49–1.84)	0.82 (0.38–1.78)
Number of vaginal examination		
0–3 times (reference)	–	–
> 3times	2.97 (1.27–3.06)	2.10 (1.21–3.65)
Place of delivery		
Health facility (reference)	–	–
Home delivery	7.88 (2.18–28.4)	9.29 (1.72–50.02)
Preterm		
Yes	2.01 (1.14–3.54)	3.15 (1.58–6.25)
Diabetes in pregnancy		
Yes	5.42 (2.19–3.42)	6.22 (1.93–20.03)
Lower abdominal pain		
Yes	2.53 (1.63–3.93)	1.99 (1.15–3.42)
Vaginal discharge		
Yes	9.10 (2.18–19.65)	7.77 (2.97–20.21)
SpO2		
< 93%	12.7 (5.56–29.08)	13.0 (4.80–37.10)
Blood glucose	0.98 (0.97–0.99)	1.01 (1.00–1.02)

**Fig. 2:**
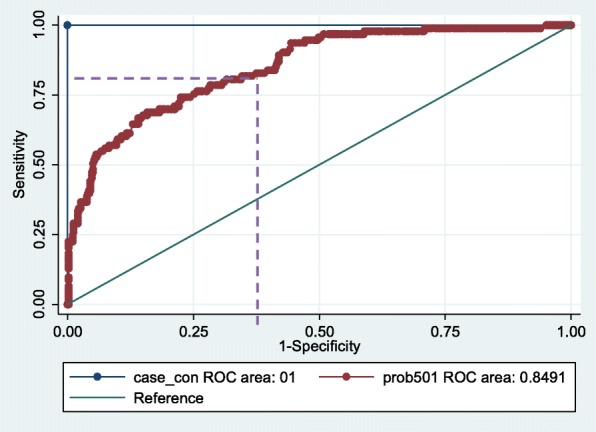
Receiver-operating characteristic curve for the clinical probability AUC = 0.84 (95% CI = 0.80–0.89)

AUC, area under the receiver operating characteristic curve was 0.84 with 95% confidence interval 0.80–0.89 which represented adequate performance (Fig. 2).

At optimal cut off of 0.069, the proposed model has 82% sensitivity and 64.8% specificity (Table 3). As sepsis is a lethal condition and involves severe consequences, it required an optimal cutoff that has less chance to miss any women with sepsis.

**Table 3:** Optimal cut offs based on probability with sensitivity and specificity

Method	Optimal Cutoff of probability	Sensitivity%	Specificity%	*PPV	*NPV
**High sensitivity and low specificity**	**0.069**	82% (73.0–88.6)	64.8% (60.4–65.9)	31.9% (28.7–35.2)	94.7% (92.1–96.4)
**Similar sensitivity and specificity**	0.086	76.3% (66.3–83.9)	74.0% (71.4–79.6)	38.7% (34.3–43.3)	94.0% (91.7–95.7)
**High specificity and low sensitivity**	0.106	70.0% (62.1–80.5)	80.7% (77.1–84.2)	43% (37.8–48.6)	93.5% (91.2–95.1)

*Positive predictive value *Negative predictive value

## Discussion

This study used to develop a model based on risk factors and clinical sign and symptoms of sepsis among postpartum women. In this study antenatal care visits, place od delivery, preterm delivery, diabetes in pregnancy, lower abdominal pain, vaginal discharge, SPO2 and blood glucose level were significant risk factors for postpartum sepsis.

Clinical and community settings both are different in terms of practice, feasibility and resource availability. As a result, models that are developed in the hospital setting may have high sensitivity and specificity but needs to be adapted accordingly to make it feasible, available and applicable for the community setting. This would enable lay health workers in the timely identification of postpartum sepsis in women and help in early referral to the tertiary care facility for management. Therefore, the next step of the study would be to conduct a validation study in the community setting and then scale it up if results are feasible.

One of the studies conducted at a tertiary care teaching hospital in Lahore which is another large city of Pakistan also provides evidence for using Score for Neonatal Acute Physiology II (SNAP II) for prediction of mortality among neonates with sepsis. The study assessed the diagnostic accuracy of SNAP II tool which includes lowest mean arterial pressure, worst PaO2/FiO2 ratio, lowest temperature, lowest serum, urine output less than 1 ml/Kg/hr. and presence of seizures. Based on the mentioned indicators severity of illness categorized into mild [1–20], moderate (21–40) and severe (> 40). SNAP II helps to identify neonates who were at high risk of mortality [12].

Socioeconomic status has been considered as an important risk factor in developing sepsis as one of the observational studies conducted at Hyderabad, Pakistan on sepsis has also recognized that women from low socioeconomic status are more prone to have sepsis [13].. Our study does not confirm socioeconomic status as a risk factor for sepsis. It is a public hospital where usually women from low or middle SES seek health care.

Previous literature highlights that hemorrhage, lacerations, multiple vaginal examination, mode of delivery are major contributors to sepsis that may develop within a few hours of giving birth [14, 15]. This study also reinforced the risk factors mentioned in previous studies and antenatal care is one of them. Antenatal Care (ANC) helps women to promote healthy home practices, health-seeking behaviors and identifies complications related to pregnancy [16, 17]. Women are more likely to give birth with a skilled birth attendant if they have had at least one ANC visit [18]. This study also depicts that not seeking antenatal care put women at a higher risk to develop sepsis. The results of this study are similar to those reported by Joseph et al. who identified that the odds of maternal deaths were 3.6 (95% CI, 1.8–7.0) times higher among those who had received no antenatal care visit [19].

Diabetes during pregnancy as significant risk factors for sepsis in this study. In sepsis, the activation of pro inflammatory indicators may lead to pathological changes that include hyperglycemia [20]. Acousta et al. explained that diabetic women had 47% greater adjusted odds of developing severe sepsis compared to septic women without diabetes [7].

Multiple vaginal examination is a contributor to infectious morbidities associated with prolonged labor. Kenyan study reported that women who had vaginal examination from 2 to 4 times and > 5 times were 2.28 and 3.8 times at higher risk of developing sepsis as compared to those women who have vaginal examination < 2 times [21]. These findings are coherent with our study as more than four hourly vaginal examinations could potentially increase the risk of sepsis due to the prolonged state of an open cervix which impairs normal mechanical barrier to infections [22].

Home delivery was a significant contributor to postpartum sepsis (aOR = 9.0; 95% CI = 1.72–50.02) in this study. A study in Pakistan reported that the odds of puerperal infection was 2.7 (95% CI; 1.1–6.2) times among women who delivered in unhygienic conditions at homes as compared to deliveries conducted at health facilities [23]. The report by State of World Children (2009) identifies that the regions with high maternal deaths have 60% of home deliveries where lack of practice of aseptic measures like hand washing, use of antiseptic materials and perinatal hygiene by unskilled birth attendants were key features for developing sepsis [24, 25]. Similar to home delivery, preterm delivery was also reported to increase the chance of sepsis by 2–3 folds [26, 27] which were also reported in this study.

Lower abdominal pain is a well-recognized non-specific symptom of puerperal sepsis. After delivery, invasion of bacteria may infect the uterus and cause pelvic inflammation which presents with lower abdominal pain [28, 29]. In this study, women with sepsis reported lower abdominal pain and vaginal discharge more commonly as compared to women without sepsis. Moreover, the odds of foul smell vaginal discharge was 3.2 times higher among women with sepsis as compared to those without.

All these pathological changes in sepsis also effect blood glucose level and blood oxygen saturation. Pulse oximetry is a non-invasive method to determine the oxygen level in the blood. In the adult population, SpO2 (> 95%) has been shown to have 90% sensitivity to detect the probability of having a pulmonary embolism [30, 31]. In SOS scoring, SpO2 had a low discriminative ability in identifying sepsis [10]. However, in this study, the contribution of SpO2 was high as it is evident by the adjusted odds ratio of 13.0 (95% CI 4.80–37.10). One of the reasons for this discrepancy may be that for SOS scoring, missing values were considered as normal so subjects with missing SpO2 values was considered as having oxygen saturation (> 95%) which ultimately make remarkable difference in results.

The Study was conducted at a tertiary level public health facility such as JPMC which caters to the population of Karachi and also receives cases from the other towns of Sindh province. The city of Karachi, being the economic hub of Pakistan have people from all over the country. Hence, the strength is that our study is generalizable to a wider population of postpartum Pakistani women. Secondly, we have used calibrated instruments for collecting information on clinical signs to reduce bias introduced by instruments.

The limitation of this study was using standard SIRS criteria for identification of cases of sepsis which itself has low sensitivity. Michael et al., found 52% (95% CI 46–58%) sensitivity of SIRS criteria for critical illness [32]. Despite this limitation, we used these diagnostic criterion because other criteria like SOS criteria or SOFA require sensitive laboratory investigations that are not routinely done in our study setting.

## Conclusion

We developed a non-invasive tool that will help identify postpartum women with sepsis as accurately as SIRS with good discriminative ability. The model revealed that women with no antenatal care, having home and preterm deliveries along with symptoms like abdominal pain, vaginal discharge and having >3vaginal examinations during labor, diabetes with high blood glucose and SpO2 less than 93% were more prone to have sepsis. The model in this study showed adequate diagnostic accuracy with high sensitivity which helps in correctly identifying woman who actually has sepsis. The model proposed in the current study used risk factors, clinical sign and symptoms, pulse oximetry and only random blood sugar test instead of any advanced laboratory investigation. Although this model requires further validation in the community-based settings to identify its applicability, it does not require highly skilled personnel for obtaining this data. This tool would be helpful in far to reach communities where front-line health workers can use it to identify high risk women and refer them to the health facility for management of sepsis and its complications, hence improving maternal outcomes. Due to differences in resource availability in remote settings, there is a dire need to identify approaches that keep in mind the feasibility and adaptability of the model based on local needs.

## Data Availability

The datasets used and/or analyzed during the current study are available from the corresponding author on reasonable request.

## Abbreviations

SIRS: systematic inflammatory response syndrome
AUC: Area under the curve
ROC: Receiver operating characteristics
PDHS: Pakistan Demographic Health Survey
SOS: Sepsis Obstetric Score
ICU: intensive care unit
JPMC: Jinnah Postgraduate Medical Center
PPH: postpartum hemorrhage
AOR: adjusted odds ratio
CI: Confidence Interval
PPV: Positive Predictive value
NPV: Negative Predictive Value
LHW: lady health workers
ANC: Antenatal Care
